# Comparison of Traditional Chinese Exercises and Nontraditional Chinese Exercise Modalities on Cognitive and Executive Function in Community Middle-Aged and Older Adults: A Cross-Sectional Study

**DOI:** 10.1155/2020/4380805

**Published:** 2020-11-22

**Authors:** Mingzhu Ye, Jian Xiong, Fang Zhao, Shanshan Sun, Lecong Wang, Guohua Zheng

**Affiliations:** ^1^Graduate School, Shanghai University of Traditional Chinese Medicine, Shanghai 201203, China; ^2^College of Nursing and Health Management, Shanghai University of Medicine & Health Sciences, Shanghai 201318, China; ^3^Shanghai University of Traditional Chinese Medicine, Shanghai 201203, China

## Abstract

**Background:**

Current evidence indicates that regular exercise can have a positive impact on cognitive function in older adults, but whether different exercise modalities may induce differential protective effects in different cognitive domains is uncertain.

**Objective:**

To compare the effect of traditional Chinese exercise (TCE) modalities and non-traditional Chinese exercise (non-TCE) modalities on cognitive and executive function in community middle-aged and older adults through a cross-sectional study.

**Methods:**

A total of 350 community middle-aged and older adults aged over 55 years participated in this study. Information on demographic characteristics, lifestyle and behavioural habits, and regular exercise was collected by a self-designed questionnaire. Global cognitive ability and executive function were assessed using the Montreal Cognitive Assessment (MoCA) scale, the clock drawing test (CDT), the animal naming test (ANT), and the trail making test (TMT). Eligible subjects were categorized into the no regular exercise (no-RE), non-TCE, or TCE groups according to their self-reported exercise information. Comparisons of global cognitive and executive function among the three groups were conducted using ANOVA or the general linear model with adjustment for potential confounding factors.

**Results:**

The results showed that for the non-TCE or TCE groups, the MoCA and CDT scores were significantly higher, and the TMT-A test time was significantly shorter than those in the no-RE group (all *P* < 0.05), but no significant difference was observed for the TMT-B and ANT tests. After adjustment for potential confounding factors, the MoCA, the CDT, and TMT-A scores in the TCE group were significant compared to those in the no-RE group. In addition, subgroup analysis showed that in the TCE group, the MoCA scores were significantly higher than those in the non-TCE group. Furthermore, in the TCE group, the CDT scores for those with an exercise duration of <5 years were higher and the TMT-A test time for those with an exercise duration of ≥5 years was shorter than those in the non-TCE group.

**Conclusions:**

Both TCE and non-TCE have potential protective effects on global cognitive and executive function in community middle-aged and older adults. Compared to the non-TCE modality, the TCE modality may have a more positive association with these protective effects. Furthermore, prospective studies are needed to confirm these findings.

## 1. Introduction

Due to increased human life expectancy, the burden of age-associated disorders or diseases will also increase exponentially in the coming decades. Cognitive disorders, or dementia, are one of the major causes of disability in the ageing population. At present, Alzheimer's disease (AD), the most dominant form of dementia, has become the fifth leading cause of death in the world and has become an important public health problem globally [1]. As an important component of cognitive function, executive function is a kind of advanced cognitive process that involves the ability to self-monitor, plan, organize, reason, be mentally flexible, and problem-solve and is usually categorized into several subdimensions, such as inhibitory control, working memory, and cognitive flexibility [2, 3]. Executive function declines with age, especially after 70, when it drops sharply [4]. The study found that execution deficit can be used as an effective indicator to distinguish those with presymptomatic AD from those who remain nondemented and participate in the process of age-related cognitive decline [5–8]. Furthermore, the decrease of early executive function state is associated with subsequent cognitive decline and is a powerful predictor of death, frailty, and disability in older adults [9]. It has been reported that the accuracy of predicting the transition from mild cognitive impairment to dementia within 3 years is as high as 86% through executive function deficit combined with language memory decline [10]. And the risk of death, frailty, and disability among older adults increased by 7%, 13%, and 11%, respectively, when the cumulative index of executive dysfunction was increased by one unit [11]. Therefore, determining the appropriate methods to enhance executive function is of great significance in slowing cognitive decline and reducing the risk of frailty and disability among the elderly.

In recent years, exercise has been increasingly recognized as an important strategy to improve cognitive and executive function. Evidence from epidemiological studies and randomized control trials demonstrated that regularly engaging in physical exercise could reduce cognitive decline at various stages of life, from younger ages to middle age and late life [12]. It has been acknowledged that physical exercise may generate substantial benefits on cognitive or executive function, but its effect mainly depends on exercise type and dose parameters (i.e., exercise intensity, frequency, and duration) [13]. It has been suggested that physical exercise with enough dose parameters (i.e., moderate-intensity aerobic exercise with a frequency of not less than 3 sessions a week and at least 20 min per session for 3 months) can contribute to the optimization of brain plasticity by triggering neurogenesis and is therefore beneficial to cognitive function [14]. A meta-analysis showed that exercise of at least moderate-intensity lasting 45–60 min per session with a frequency of 3 sessions per week for at least 3 months can improve global cognition, attention, and executive function in healthy adults over 50. Exercise type refers to the modality of exercise training, and the most common modalities of exercise training are aerobic training, resistance training, and multicomponent training (incorporating both aerobic and resistance training). Previous studies have shown that aerobic exercise, resistance training, or multicomponent training with enough exercise volume would have a positive association with improvement in cognitive or executive performance for older individuals with/without cognitive impairment [15]. For example, Dustman RE et al. reported that after aerobic exercise intervention three times per week for four months, the executive function of older sedentary adults was significantly improved [16]. Liu-Ambrose T and Nagamatsu L S' also showed that resistance exercise once or twice a week for 60 min and lasting for 12 months was associated with the executive function of elderly women [17]. However, Young J, Snowden M and other scholars conducted a systematic evaluation of previous studies and found that there was not enough evidence to prove that exercise could improve cognitive and executive function [18, 19]. As special aerobic exercises, traditional Chinese exercises emphasize the importance of the harmony and unity of the human essence, qi, and spirit by regulating spirit, breath, and mind while performing physical exercise. These are mind-body exercises aimed at creating effective positive interactions among the brain, mind, and body [20, 21]. Several systematic studies have suggested that traditional Chinese exercise (TCE) (i.e., Baduanjin and Tai Chi) has positive effects on the cognitive and executive function (i.e., inhibition control and cognitive flexibility) of older adults [22–24]. However, not all meta-analyses found this positive effect in older adults. Zhang Q and Hu J examined 5 studies of TCE interventions ranging from 12 to 24 weeks and found that TCE promoted improvement of visuospatial function, not global cognitive or executive function, in older adults [25]. Therefore, the exercise modality has been utilized in different studies with varying effects, but the improvement of cognitive and executive function with different exercise modalities remains uncertain [26]. The present study, therefore, aimed to investigate the influence of different exercise modalities on cognitive and executive function for middle-aged and older people in the community.

## 2. Materials and Methods

### 2.1. Study Population

This was a cross-sectional study. A total of 350 subjects were recruited by a free clinic and via word of mouth from the communities in Yuchen County, Henan Province, China, from 10 July 2018 to 31 August 2018. Written informed consent was obtained from all participants before data collection. This study was approved by the ethics committee of Shanghai University of Medicine and Health Sciences. Community middle-aged and older adults aged over 55 years were included in this study, but those who met the following conditions, which were assessed based on medical records, were excluded: ① suffering from diseases or disorders that affect cognitive and executive functions (such as depression, schizophrenia, dementia, stroke, or epilepsy), ② MoCA scale score <15, and ③ suffering from motor dysfunction (i.e., movement vertigo and exercise anaemia). Forty-six subjects were excluded because they met the exclusion criteria. Ultimately, a total of 304 subjects were included in the final analysis.

### 2.2. Investigation of Demographic Characteristics and Lifestyle and Behavioural Habits

The demographic characteristics and lifestyle and behavioural habits of the participants were investigated using a self-designed questionnaire. The questionnaire included the following components: ① sociodemographic characteristics (age, gender, height, weight, education, marital status, occupation, and educational status); ② lifestyle and behavioural habits (smoking, drinking, sleep status, taste preferences, social activities, and physical activity level); and ③ physical condition (whether suffering from diabetes or hypertension). The above data were obtained by participant self-report, and body mass index (BMI) was calculated based on height and weight.

### 2.3. Cognitive Assessment

Global cognitive function was assessed using the MoCA scale (Beijing version), which contains 8 items addressing visuospatial/executive function, naming, memory, attention, language, abstraction, delayed recall, and orientation. If the subject's length of education was ≤12 years, the total score on the test included an additional point. The highest score was 30 points, and higher scores indicated higher global cognitive function [27].

Executive function and its subdimensions (i.e., working memory, cognitive flexibility, and inhibitory control) were assessed using the clock drawing test (CDT), the animal naming test (ANT), and the trail making test (TMT). The CDT was used to measure executive function. In the experiment adopting the CDT four-point method, subjects were asked to draw a closed circle; 12 Arabic numerals were marked on the circle; the numbers had the correct position and order, and the pointer was placed at the position of 11 : 10. One point was awarded for each correct action, with a maximum score of 4 points [28]. Inhibitory control and working memory were assessed using the ANT: the subjects were required to name as many animals as possible within 60 s, with 1 point awarded for each kind of animal and repeated animals earning no points [29]. TMT were used to assess cognitive flexibility. This test consists of two parts (i.e., TMT-A and TMT-B) [30]. The TMT-A requires the subjects to connect the numbers 1–25 in order from smallest to largest with a line as quickly as possible. The TMT-B requires the subjects to accurately switch between numbers and letters as quickly as possible. Ultimately, statisticians recorded the time in seconds required for the subjects to complete the TMT-A and TMT-B tests, and a longer time indicated greater impairment [31, 32].

The above tests were administered by strictly trained researchers using paper versions.

### 2.4. Categorization of Exercise Modality

The exercise modality was investigated by self-designed questionnaires, which included the following information: regular exercise or not, type of exercise, and duration and frequency of exercise. Here, we defined regular exercise as engagement in any type of exercise for at least three months with a frequency of at least three sessions per week and at least twenty minutes per section [14, 33]. Subjects were divided into three groups according to their reported exercise information: (1) TCE group: regularly performing one or more traditional Chinese exercises (such as Tai Chi, Baduanjin, Wuqinxi, or Yijinjing); (2) nontraditional Chinese exercise (non-TCE) group: regularly performing one or a variety of nontraditional Chinese exercises (e.g., walking, aerobic running, or square dancing); and (3) no regular exercise (no-RE) group: not regularly engaged in a certain type of exercise for at least three months, or exercise volume not meeting the criteria of regular exercise.

### 2.5. Statistical Analysis

All statistical analyses were conducted by SPSS 24.0 software, and a 2-sided *P* < 0.05 was considered to be statistically significant. The missing data were filled in using the multiple imputation method. The means and standard deviations (SDs) were used to present a summary of the continuous variables, and frequencies and percentages were used for the categorical variables. The demographic characteristics and lifestyle and behavioural habits among the three groups were analysed using one-way ANOVA or the Kruskal–Wallis test for continuous variables and the chi-square test for categorical variables. Comparisons of cognitive and executive function among the three groups were performed using a one-way ANOVA or the Kruskal–Wallis test, and post hoc tests were used for multiple comparisons if group differences were examined. To address the adjusted effect, generalized linear models (GLMs) were constructed, with cognitive or executive function score as the dependent variable, exercise modality as the independent variable, and demographic factors or other bias factors as covariates. In addition, subgroup analysis for cognitive and executive function between the TCE and non-TCE groups was performed using a 2-sample *t*-test according to the exercise duration.

## 3. Results

### 3.1. Sample Characteristics

Among 304 eligible subjects, 94 (30.92%) were categorized into the no-RE group, 119 (39.14%) into the non-TCE group, and 91 (29.94%) into the TCE group. The mean age of the participants was 66.7 (SD = 7.46) years, with ages ranging from 55 to 87 years. The comparison of basic characteristics among the three groups is shown in Table 1. Significant differences among groups were observed for gender, the education level, occupation, smoking, and the physical activity level (*P* < 0.05).

### 3.2. Comparison of Cognitive and Executive Function among Groups

Comparisons of cognitive and executive function among the three groups are shown in Table 2. Significant differences in MoCA scores, CDT scores, and the TMT-A test among the three groups were found (*P* < 0.05). In the two exercise groups, MoCA and CDT scores were significantly higher, and TMT-A test times were significantly shorter than in the no-RE group (all *P* < 0.05), while the MoCA and CDT scores in the TCE group were significantly higher than those in the non-TCE group (*P* < 0.05). There were no significant differences in ANT scores or TMT-B test times among the three groups.

A significant association was observed between the non-TCE group and MoCA and CDT, and between the TCE group and MoCA, CDT, and TMT-A by using multivariate general model analysis. We included a variety of potential confounders in the model. However, the results did not change substantially even after adjustment for these confounders except for the non-TCE group. In the crude model, we observed a significant association between the non-TCE group and MoCA scores and CDT scores, even after adjustment for gender and the education level, but after adjustments were made for all potential confounders, this association became nonsignificant. In the final model, compared to no-RE, regular TCE may improve MoCA scores by 2.28 (95% CI:1.28–3.28), improve CDT scores by 0.72 (95% CI: 0.32–1.12), and shorten TMT-A times by 17.91 s (95% CI: 5.43–30.38) (Table 3).

### 3.3. Comparison of Cognitive and Executive Function between the Non-TCE Group and TCE Group

We conducted a stratified analysis between the non-TCE and TCE groups according to their exercise duration (Table 4). The MoCA scores in the TCE group were significantly higher than those in the non-TCE group regardless of total years of exercise (i.e., less than 5 years or over 5 years). The CDT scores of the TCE group were higher than those of the non-TCE group when the exercise duration was less than 5 years, but no significant difference was found when the exercise duration was over 5 years. The TMT-A test time in the TCE group was shorter than that in the non-TCE group when the exercise duration was over 5 years. After stratified analysis, there were no significant differences in AND and TMT-B between the non-TCE and TCE groups.

## 4. Discussion

This is the first study to observe the effect of different exercise modalities on cognitive and executive function in community middle-aged and older people. The results of the present study showed that compared with no-RE, TCE can increase MoCA scores and CDT scores and reduce TMT-A test times for community middle-aged and older adults. Furthermore, compared with the non-TCE group, the TCE group had higher MoCA and CDT scores and shorter TMT-A test times. The findings suggest that TCE can improve global cognitive ability, executive function, and more subdimensions of executive function for middle-aged and older adults in the community. TCE was superior to non-TCE in improving global cognitive ability and some subdimensions of executive function.

It is well known that physical activity or exercise can have a positive impact on cognitive function and the possible mechanism associated with the processes of neurogenesis, vascularization, and increased blood flow in the brain, as well as changes in the secreted levels of some biomarkers in the neurochemical system [34]. However, different exercise modalities may impact different cognitive functions and distinct brain regions [35]. Aerobic exercise induces metabolic, respiratory, and cardiovascular changes in the body, while resistance training affects metabolic and energetic processes and, to some extent, intramuscular coordination [36]. Although mind-body exercise may induce less energy metabolism than aerobic or resistance training, it emphasizes coordination of the body and mind, including the breath, body movement, and mind, which require perceptual and higher-level cognitive processes, such as attention and executive ability. Thus, changes induced by TCE are likely to be related to changes in cognitive processing [22–24]. The current investigation classified community middle-aged and older adults engaging in regular exercise into either the TCE group or the non-TCE group according to the type of exercise they practised. In the non-TCE group, the exercise type practised was mainly aerobic exercise, such as walking, aerobic running, and square dancing. This study found that aerobic exercise could have a positive benefit on global cognitive ability and CDT test performance, compared with no-RE training, but this positive effect was not significant after adjustments were made for confounders, such as gender and education. These findings suggest that non-TCE may have a weak beneficial effect on the global cognitive ability and executive function of community middle-aged and older adults. Several systematic reviews and meta-analyses supported that regular aerobic exercises could improve global cognitive function in older adults with cognitive impairment [37, 38]. However, this effect is controversial for older adults without cognitive impairment. A Cochrane systematic review from 12 randomized controlled trials did not find a beneficial effect of aerobic exercise on any cognitive function, including global cognitive ability, memory, and executive function, in cognitively healthy older adults [18]. However, a recent study in rodents showed that aerobic exercise could improve spatial working memory and hippocampal plasticity in ageing rats via the molecular mechanisms of increased glutamatergic signalling and reduced DNA damage [39]. Another review indicated that moderate-intensity aerobic exercise practised for at least 6 months may have a protective effect on the cognitive function of healthy older adults [40]. Therefore, an increasing number of studies are needed to confirm whether aerobic training improves cognition in healthy middle-aged and older adults. In this study, we found that the TCE could increase MoCA and CDT scores and lower TMT-A test times in community middle-aged and older adults even after adjustment for several confounding factors, including sex, education, occupation, smoking, and the physical activity level, but no significant improvements were found for the ANT and TMT-B. These findings may indicate that TCE could have a protective effect on global cognitive ability and a positive benefit on the executive function of community middle-aged and older adults. Our findings are in line with previous studies. At least four systematic reviews reported that TCEs such as Tai Chi and Baduanjin showed positive protective effects on global cognitive ability and executive function in healthy middle-aged or older adults [23–25, 41]. TCEs such as Tai Chi and Baduanjin are complex holistic mind-body exercises with the obvious characteristics of traditional Chinese medicine, which involves the cooperation of physical postures and movement, breathing, and relaxation [42]. Most TCEs consist of many complex postures and movements, such as Tai Chi exercise, which includes 24 forms, 48 forms, or 108 forms. Therefore, they demand more attention, execution, and memory throughout their practice, as well as high-level balance control and fast reaction time, which in turn could facilitate further cognitive processing such as memory and executive function. The relaxation effect of TCEs may also play a part in promoting cognitive and executive function [43]. In this study, our subanalysis showed that the MoCA scores in the TCE group were higher than those in the non-TCE group regardless of whether the exercise duration was less than 5 years or more than 5 years, and this finding suggests that TCE may be superior to non-TCE in improving the global cognitive ability of community middle-aged and older adults. This superiority in cognitive function may be related to the exercise characteristics of TCE. In addition, we also found that the CDT scores in the TCE group were higher than those in the non-TCE group when the exercise duration was less than 5 years, while the TMT-A test time was shorter than that in the non-TCE group when the exercise duration was more than 5 years. The CDT was originally developed to assess visuo-constructional ability, and it is reliable to explore executive functions because of the demands for visuospatial attention, motor skills, conceptualization, and planning [44]. The TMT test, including part A and part B, is a well-established test sensitive to impairment in multiple cognitive domains. This test requires coordination of motor speed and agility, and its performance of TMT-B is more indicative of executive function, especially cognitive flexibility of execution, than TMT-A [45]. The current study showed that the performances of both the TMT-A and TMT-B tests in the TCE group were better than those in the non-TCE group, but only the difference between groups on the TMT-A test at exercise durations over 5 years was significant. These findings indicate that TCE may have a better effect on the executive function of community middle-aged and older adults than non-TCE.

Several limitations should be considered in the interpretation of our findings. First, this is a cross-sectional study, and most of the data obtained were based on self-report responses; thus, no causal relationships can be unambiguously established. Second, the cross-sectional study with an observational design does not allow us to control the exercise volume for participants, which is a key factor in evaluating the effect of different exercise modes on cognitive ability. The type, frequency, and duration of exercise in this study mainly depend on the self-report of participants; therefore, recall bias from participants is inevitable. Finally, although we adjusted for the influence of many potential confounders, we also did not exclude the confounding possibility of the uninvestigated factors. These methodological limitations warrant caution in interpreting results and exemplify the need for prospective studies in this research area.

## 5. Conclusion

The results of this study suggest that regular non-TCE or TCE is potentially beneficial to protect global cognitive or executive function decline among community middle-aged and older adults, while TCE is more positively associated with this protective effect of global cognitive ability and executive function than non-TCE. However, prospective studies are needed to further confirm these findings.

## Figures and Tables

**Table 1:** Sample demographics and clinical characteristics.

Characteristic	No-RE group (*n* = 94)	Non-TCE group (*n* = 119)	TCE group (*n* = 91)	(*F*/*χ*^2^) value	*P* value
Age (years), mean (SD)	67.29 ± 7.69	66.40 ± 7.47	66.30 ± 7.26	0.508	0.602
Sex, *n* (%)					
Male	39 (41.5%)	32 (26.9%)	42 (46.2%)		
Female	55 (58.5%)	87 (73.1%)	49 (53.8%)	9.280	0.010^*∗*^
Education (years), *n* (%)					
≤12	92 (97.9%)	107 (89.9%)	69 (75.8%)		
>12	2 (2.1%)	12 (10.1%)	22 (24.2%)	22.109	<0.001^*∗*^
Occupation, *n* (%)					
Physical labour	66 (70.2%)	54 (45.4%)	34 (37.4%)		
Mental labour	28 (29.8%)	65 (54.6%)	57 (62.6%)	22.143	<0.001^*∗*^
BMI (kg/m^2^), mean (SD)^#^	24.61 ± 3.62	25.06 ± 3.15	24.44 ± 2.83	1.068	0.345
Marital status, *n* (%)					
Married	76 (80.9%)	97 (81.5%)	80 (87.9%)		
Others	18 (19.1%)	22 (18.5%)	11 (12.1%)	2.061	0.357
Smoking, *n* (%)					
Never	66 (70.2%)	105 (88.2%)	73 (80.2%)		
Former	16 (17.0%)	9 (7.6%)	11 (12.1%)		
Current	12 (12.8%)	5 (4.2%)	7 (7.7%)	10.985	0.027^*∗*^
Drinking, *n* (%)					
No	73 (77.7%)	99 (83.2%)	64 (70.3%)		
Yes	21 (22.3%)	20 (16.8%)	27 (29.7%)	4.914	0.086
Taste preferences, *n* (%)					
Heavy	23 (24.5%)	22 (18.5%)	19 (20.8%)		
Normal	38 (40.4%)	42 (35.3%)	37 (40.7%)		
Bland	33 (35.1%)	55 (46.2%)	35 (38.5%)	3.144	0.534
Sleep status (hours), *n* (%)					
≤6	63 (67.0%)	78 (65.5%)	53 (58.2%)		
>6	31 (33.0%)	41 (34.5%)	38 (41.8%)	1.797	0.407
Social activities, *n* (%)					
No	11 (11.7%)	16 (13.4%)	8 (8.8%)		
Yes	83 (88.3%)	103 (86.6%)	83 (91.2%)	1.101	0.577
Chronic diseases, *n* (%)					
None	79 (84.1%)	94 (79.0%)	75 (82.4%)		
Hypertension	5 (5.3%)	12 (10.1%)	8 (8.8%)		
Diabetes	2 (2.1%)	2 (1.7%)	1 (1.1%)		
Others	8 (8.5%)	11 (9.2%)	7 (7.7%)	2.121	0.908
Physical activity level, *n* (%)					
Low level	34 (36.2%)	8 (6.7%)	13 (14.3%)		
Medium level	54 (57.4%)	70 (58.8%)	54 (59.3%)		
High level	6 (6.4%)	41 (34.5%)	24 (26.4%)	44.467	<0.001^*∗*^

No-RE, no regular exercise; non-TCE, nontraditional Chinese exercise; TCE, traditional Chinese exercise; SD, standard deviation; BMI, body mass index. ^#^Having missing value; ^*∗*^*P* < 0.05.

**Table 2:** Comparison of cognitive and executive function in three groups (mean ± SD).

	No-RE group (*n* = 94)	Non-TCE group (*n* = 119)	TCE group (*n* = 91)	*F* value	*P* value
MoCA (scores)	21.22 ± 3.56	22.52 ± 3.54^*a*^	24.45 ± 2.68^*a*,*b*^	22.267	<0.001^*∗∗*^
CDT (scores)	2.48 ± 1.47	2.93 ± 1.26^*a*^	3.38 ± 1.09^*a*,*b*^	11.504	<0.001^*∗∗*^
ANT (scores)	17.73 ± 3.72	17.99 ± 4.47	18.49 ± 4.11	0.811	0.445
TMT-A (s)	91.00 ± 45.33	85.36 ± 46.27^*a*^	66.89 ± 25.69^*a*^	8.878	<0.001^*∗∗*^
TMT-B (s)	118.37 ± 70.04	118.05 ± 71.75	110.90 ± 58.85	0.371	0.690

^*a*^ *P* < 0.05 compared to the no-RE group; ^*b*^*P* < 0.05 compared to the non-TCE group; ^*∗*^*P* < 0.05; ^*∗∗*^*P* < 0.01.

**Table 3:** Associations between exercise type and cognitive and executive function from the generalized linear models analysis.

	No-RE group	Non-TCE group	TCE group
	B (95% CI)	B (95% CI)	B (95% CI)
MOCA			
Model 1	Reference	1.30 (0.40, 2.19)^*∗∗*^	3.23 (2.27, 4.19)^*∗∗*^
Model 2	Reference	1.20 (0.30, 2.10)^*∗∗*^	2.96 (1.97, 3.95)^*∗∗*^
Model 3	Reference	1.31 (0.40, 2.21)^*∗∗*^	2.94 (1.95, 3.93)^*∗∗*^
Model 4	Reference	0.87 (−0.02, 1.75)	2.50 (1.54, 3.46)^*∗∗*^
Model 5	Reference	0.85 (−0.05, 1.74)	2.48 (1.51, 3.45)^*∗∗*^
Model 6	Reference	0.56 (−0.39, 1.52)	2.28 (1.28, 3.28)^*∗∗*^

CDT			
Model 1	Reference	0.45 (0.11, 0.80)^*∗*^	0.91 (0.53, 1.28)^*∗∗*^
Model 2	Reference	0.44 (0.18, 0.87)^*∗*^	0.87 (0.49, 1.26)^*∗∗*^
Model 3	Reference	0.51 (0.16, 0.86)^*∗∗*^	0.86 (0.48, 1.24)^*∗∗*^
Model 4	Reference	0.45 (0.10, 0.81)^*∗*^	0.80 (0.41, 1.18)^*∗∗*^
Model 5	Reference	0.43 (0.07, 0.78)^*∗*^	0.77 (0.39, 1.16)^*∗∗*^
Model 6	Reference	0.35 (−0.03,0.74)	0.72 (0.32, 1.12)^*∗∗*^

ANT			
Model 1	Reference	0.25 (−0.87, 1.38)	0.76 (−0.44, 1.96)
Model 2	Reference	0.90 (−1.03, 1.21)	0.31 (−0.92, 1.54)
Model 3	Reference	0.16 (−0.97, 1.29)	0.30 (−0.93, 1.53)
Model 4	Reference	0.08 (−1.07, 1.23)	0.22 (−1.03, 1.47)
Model 5	Reference	0.13 (−1.04, 1.29)	0.26 (−1.00, 1.52)
Model 6	Reference	0.45 (−0.80, 1.69)	0.49 (−0.81, 1.79)

TMT-A (s)			
Model 1	Reference	−5.64 (−16.74, 5.46)	−24.11 (−35.94, −12.28)^*∗∗*^
Model 2	Reference	−4.09 (−15.15, 6.97)	−19.81 (−31.97, −7.65)^*∗∗*^
Model 3	Reference	−5.66 (−16.75, 5.44)	−19.55 (−31.64, −7.46)^*∗∗*^
Model 4	Reference	−2.39 (−13.53, 8.76)	−16.28 (-28.40, −4.16)^*∗∗*^
Model 5	Reference	−4.53 (−15.69, 6.62)	−18.25 (−30.34, −6.16)^*∗∗*^
Model 6	Reference	−4.05 (−16.01, 7.90)	−17.91 (−30.38, −5.43)^*∗∗*^

TMT-B (s)			
Model 1	Reference	−0.33 (−18.68, 18.03)	−7.47 (−27.03, 12.09)
Model 2	Reference	0.95 (−17.49, 19.39)	−3.94 (−24.22, 16.33)
Model 3	Reference	0.24 (−18.38, 18.87)	−3.83 (−24.13, 16.48)
Model 4	Reference	3.69 (−15.21, 22.58)	−0.39 (−20.92, 20.14)
Model 5	Reference	0.40 (−18.54, 19.35)	−3.40 (−23.92, 17.13)
Model 6	Reference	2.62 (−17.67, 22.91)	−1.82 (−23.00, 19.35)

Model 1, unadjusted; model 2, adjusted for duration of education; model 3, adjusted for model 2 + gender; model 4, adjusted for model 3 + occupation; model 5, adjusted for model 4 + smoking; model 6, adjusted for model 5 + physical activity level. ^*∗*^*P* < 0.05; ^*∗∗*^*P* < 0.01.

**Table 4:** Comparison of cognitive and executive function between the non-TCE group and TCE group.

	Non-TCE group (*n* = 119)	TCE group (*n* = 91)	*t* value	*P* value
<5 years				
*n*	52	17		
MoCA (scores), mean (SD)	22.28 ± 3.81	25.30 ± 2.38	3.074	0.003^*∗∗*^
CDT (scores), mean (SD)	2.77 ± 1.32	3.65 ± 0.49	4.010	<0.001^*∗∗*^
ANT (scores), mean (SD)	17.68 ± 4.22	18.59 ± 3.91	0.784	0.436
TMT-A (s), mean (SD)	85.86 ± 48.13	61.99 ± 27.27	−1.939	0.057
TMT-B (s), mean (SD)	107.53 ± 74.36	104.55 ± 48.73	−0.154	0.878

≥5 years				
*n*	67	74		
MoCA (scores), mean (SD)	22.70 ± 3.34	24.26 ± 2.72	3.012	0.003^*∗∗*^
CDT (scores), mean (SD)	3.06 ± 1.21	3.32 ± 1.18	1.315	0.191
ANT (scores), mean (SD)	18.22 ± 4.67	18.47 ± 4.18	0.334	0.739
TMT-A (s), mean (SD)	84.97 ± 45.14	68.01 ± 25.37	−2.712	0.008^*∗∗*^
TMT-B (s), mean (SD)	126.21 ± 69.11	112.36 ± 61.13	−1.262	0.209

^*∗*^ *P* < 0.05; ^*∗∗*^*P* < 0.01.

## Data Availability

The data used to support the findings of this study are available from the first author upon request.

## Abbreviations

TCE: Traditional Chinese exercise
Non-TCE: Nontraditional Chinese exercise
No-RE: No regular exercise
MoCA: Montreal cognitive assessment
CDT: Clock drawing test
ANT: Animal naming test
TMT: Trial making test
AD: Alzheimer's disease
SD: Standard deviations
GLMs: Generalized linear models
BMI: Body mass index.
